# Common Symptoms of Mild Traumatic Brain Injury and Work Functioning of Active-Duty Service Members with a History of Deployment

**DOI:** 10.3390/ijerph18158079

**Published:** 2021-07-30

**Authors:** Patrick Richard, Nilam Patel, Daniel Gedeon, Regine Hyppolite, Mustafa Younis

**Affiliations:** 1 Department of Preventive Medicine and Biostatistics, Uniformed Services University of the Health Sciences, Bethesda, MD 20814, USA; 2 The Henry M. Jackson Foundation for the Advancement of Military Medicine, Bethesda, MD 20814, USA; nilam.patel.ctr@usuhs.edu (N.P.); daniel.gedeon.ctr@usuhs.edu (D.G.); regine.hyppolite.ctr@usuhs.edu (R.H.); 3 Department of Health Policy and Management, School of Public Health, Jackson State University, Jackson, MS 39217, USA; younis99@gmail.com

**Keywords:** work functioning, mild traumatic brain injury, active-duty service members, Military Health System

## Abstract

This study used data from the Military Health System Data Repository to examine the association between mild traumatic brain injuries (mTBI) and work functioning such as work duty limitations, hospital emergency room visits and inpatient admissions for active-duty service members (ADSMs). Further, this study assessed the role that common symptoms of mTBI play in work functioning. Multivariate results showed that having a mTBI diagnosis is not a major factor that results in being “released with work duty limitations”. However, findings from these regression models also showed that the interaction of mTBI with cognitive and linguistic symptoms resulted in odds of 3.63 (CI: 1.40–9.36, *p* < 0.01) for being “released with work duty limitations” and odds of 4.98 (CI: 1.16–21.39, *p* < 0.05) for having any emergency department visits compared to those with no diagnosis of mTBI and none of these symptoms. Additionally, the interaction of mTBI with sleep disturbance and chronic pain showed odds of 2.72 (CI: 1.31–5.65, *p* < 0.01) and odds of 11.56 (CI: 2.65–50.44, *p* < 0.01) for being “released with work duty limitations” compared to those with no diagnosis of TBI and none of these symptoms, respectively. Further research is needed to investigate the association between mTBI and duration of time off work to provide a comprehensive understanding of the effect of mTBI on work functioning in the Military Health System.

## 1. Introduction

The purpose of this study is to examine the association between mild traumatic brain injuries (mTBI) and work functioning for active-duty service members (ADSMs) with a history of deployment. Work functioning is defined as ADSMs who returned to duty post-injury but have work duty limitations, have any hospital emergency room visits, inpatient admissions and length of stay for those with any hospital admissions. Although ADSMs who experience work duty limitations or use emergency rooms or inpatient hospital services are not restricted from work participation, these factors still represent an important workplace limitation in terms of readiness as they are not “deployable” and take frequent time off work. Hence, the concept of work functioning here is consistent with the conceptual framework being proposed by Sandqvist and Henriksson [1]. In this framework, the authors defined work functioning as an intersection between work participation, work performance, and individual capacity. This study also assesses the role that common symptoms of mTBI play in work functioning. Using the Traumatic Brain Injury Center of Excellence (formerly the Defense and Veterans Brain Injury Center) classification, the common symptoms of mTBI are grouped into: cognitive/linguistic, hearing, neurologic, emotional/behavioral, sleep, vision and other categories of disturbances [2].

From a human capital perspective, there are both benefits and sacrifices of military service, which are also associated with work functioning for ADSMs. On the one hand, ADSMs have greater opportunities to enhance their human capital through education and training programs while in the military [3,4]. On the other hand, while service members enter the military in relatively better health than their civilian counterparts [5], they face unique threats, particularly during deployment, that may adversely impact their health and hence, their ability to return to duty or the quality of work for those who return to duty [6,7]. Indeed, traumatic brain injuries (TBI) have increased significantly among ADSMs during the past decade because of Operation Enduring Freedom/Operation Iraqi Freedom, and TBI has been considered the “signature” injury of these wars [8]. Incidence rates of TBI of about 11% to 23% have been found in those who participated in these wars [9,10,11,12,13]. Subjects of these studies are similar to those in our study as our focus is on the population of ADSMs who have had a history of deployment to Operation Enduring Freedom/Operation Iraqi Freedom [8,9,10,11,12,13].

There is an emerging body of literature investigating the relationship between deployment-related TBI and employment outcomes as well as the identification of some of the factors that are associated with these outcomes [5,7,14,15,16,17,18,19,20,21,22,23,24,25,26,27]. However, much of this literature focuses on assessing vocational outcomes such as employment status and short- and long-term employment stability among veterans diagnosed with moderate or severe TBI receiving rehabilitative services or participating in employment-oriented programs [5,7,14,15,16,17,18,19,20,21,22,23,24,25,26,27]. Most of these patients suffer from “polytrauma” such as a traumatic limb loss and chronic physical health conditions resulting mostly from multiple blast explosives during deployment. TBI in conjunction with polytrauma have different sequelae and show unique pathophysiological complexity. These patients usually experience a number of cognitive, emotional and interpersonal sequelae such as impaired decision-making and problem-solving skills that may adversely and significantly impact their employment outcomes.

While it is important to understand the relationship between TBI-related impairments and employment outcomes for veterans, it is also important to understand the effect of TBI experienced during deployment on the work functioning of ADSMs diagnosed with mTBI. Indeed, recent data from the Defense and Veterans Brain Injury Center show that about 417,503 ADSMs have been diagnosed with combat-related TBI between 2000 and 2019 [2] and the majority of them, over 82%, suffer from mTBI. Studies have found that about 76% of individuals diagnosed with mTBI returned to work within six months after the injury and about 79% returned to work one year after the injury [14,28,29,30,31]. However, it is surprising that there is such a paucity of studies assessing how these patients function once they return to work or duty, despite the high number of ADSMs diagnosed with mTBI that have returned to the workplace. Even after returning to work or duty, patients diagnosed with mTBI often experience common symptoms such as sleep disturbances, headaches, depression, alcohol abuse/dependence, and post-traumatic stress disorder (PTSD) that may impact their work functioning [14,29,30,31].

Relevant to our analysis, there was a presentation by Bauer et al. that examined the relationship between Army ADSMs with a history of deployment and diagnosed with mTBI and other comorbid conditions such as behavioral health problems and chronic pain that had military duty limitations, among other outcomes [32]. Another paper by Larson and colleagues [33] found that Army soldiers who received early nonpharmacological treatment modalities had lower odds of being put on military duty limitations as a result of an encounter with a healthcare provider. On the other hand, soldiers who were prescribed only opioids either at an index visit or during a follow-up visit had higher odds of being put on military duty limitations as a result of an encounter with a healthcare provider. While findings from these studies are informative for our analysis, our study expands this literature to include other measures of work functioning such as emergency department (ED) visits and inpatient admissions. More importantly and based on the Defense and Veterans Brain Injury Center framework, which has important implications for clinical guidelines and treatment in the Department of Defense and veterans affairs, we hypothesized that common symptoms of mTBI including cognitive/linguistic, hearing, neurologic, emotional/behavioral, sleep disturbances, vision and other symptoms such as nausea, vomiting, headache, neuralgia, neuritis, other malaise, and chronic and other fatigue are the key mechanisms through which mTBI would impact work functioning. A diagnosis of mTBI may impact patients’ work functioning in a number of ways. In our conceptual framework, we postulate that common symptoms of mTBI are the key mechanisms through which mTBI would impact work functioning. The more common these symptoms are, the more ADSM patients with mTBI are likely to use emergency room or hospital inpatient services, hence disrupting their work functioning or military readiness. Additionally, building on the bodies of literature on civilian and veteran affairs on this topic that show that factors such as age, age at injury, sex, race/ethnicity, education, previous employment or occupation, marital status, preinjury substance misuse, and days of post-traumatic amnesia are associated with short- and long-term employment outcomes for patients with mTBI, we controlled for most of these factors in our study analysis.

To our knowledge, this study is the first to capitalize on the unique opportunity offered by the Military Health System Data Repository (MDR) to assess the relationship between mTBI and work functioning of ADSMs. Findings from this study may have important implications for both military and civilian populations by refining our understanding of the mechanisms through which these common symptoms of mTBI may be associated with work functioning, which has important implications for military readiness, quality of life and rehabilitative services.

## 2. Materials and Methods

### 2.1. Participants

#### 2.1.1. Data Sources and Study Population

This study used retrospective outpatient and inpatient data from the MDR from 2015 to 2019 for 2671 patients who were 18–64 years old and diagnosed with mTBI and 20,039 matched controls using a propensity score approach (total sample = 22,710 ADSMs with a history of deployment). We focused on patients receiving direct care only because the outcomes under study such as “released with work duty limitations” are captured for these patients only. MDR has administrative claims data that contain coverage, workload, deployment, demographic, clinical information on every encounter whether at a Military Treatment Facility, civilian clinic, or a hospital by all active-duty personnel, military retirees and their families in the United States and abroad. Data on all beneficiaries using care in the MDR include diagnostic codes related to the condition (s) or reason (s) for receiving care and procedure codes associated with any procedures performed during either inpatient or outpatient visits. These diagnoses and inpatient procedures are coded using the International Classification of Diseases, Tenth Revision, Clinical Modification (ICD-10-CM) and outpatient procedures are coded using Current Procedural Terminology codes.

#### 2.1.2. Study Subjects

There were 23,470 ADSMs with a history of deployment in the MDR from 2015 to 2019. We excluded those who were currently deployed and those who were diagnosed with moderate and severe TBI to arrive at a sample of 22,914 subjects with no missing observations. A major challenge in the use of observational and population-based data is that there are systematic differences in the characteristics of those with the condition (mTBI) compared to those without the condition (no TBI), which may bias or confound the results. Even after controlling for an extensive set of covariates observed in the data by using multivariate models, bias may still remain [25,26,27]. To address this issue, we used a propensity score matching approach to balance the sample characteristics between the two groups (mTBI vs. no TBI) using all of the available socio-demographic, clinical, behavioral and common symptoms of TBI [34,35]. In this case, a propensity score is the probability or a balancing score that is computed using a logistic regression model with mTBI as the dependent variable while controlling for all the covariates in Table 1 and Table 2 [35]. This resulted in a balanced analytic sample of 22,710 ADSMs. We excluded National Guard and Reserve members from the analysis because the MDR may not have complete data for these service members, particularly if they use their private insurance to receive care elsewhere.

### 2.2. Measures

#### 2.2.1. Dependent Variables and Key Independent Variable

Three primary binary outcomes, coded as 1 if yes or 0 otherwise, were used to measure work functioning: (1) released with work duty limitations; (2) any ED visits; and (3) any hospital inpatient admissions. A secondary outcome was the length of stay for a subset of patients (*n* = 696) with any hospital inpatient admissions. mTBI was the key independent variable and was measured using the Department of Defense Unique Codes (DOD0102) based on the recommendation from the Traumatic Brain Injury Center of Excellence [2]. This Center recommended using a specific Department of Defense code that is based on the Glasgow coma scale scores 13–15 to measure mTBI. The Glasgow coma scale is a neurological scale that measures the level of consciousness and consists of three components—eye opening, verbal response and motor response [36,37,38]. The total score from these components ranges from 3 to 15, with lower scores being more severe. mTBI is usually an asymptomatic and isolated head injury with a Glasgow coma scale score ranging from 13 to 15.

#### 2.2.2. Covariates

The models controlled for an extensive set of covariates based on the human capital model augmented by the literature on this topic [14,29,30,31]. These covariates included age, sex, race, marital status, military pay grade or rank, branch of service, geographic market areas and time trend effects. In terms of age, a square term was added to the model to capture the non-linear association between age and the dependent variables. Geographic market area variables based on the Military Health System (MHS) were included to account for geographic variances in the diagnostic and clinical decision making in TBI-related outcomes. Race and ethnicity were divided into six categories of White (non-Hispanic), Black (non-Hispanic), Hispanic, Asian and Pacific Islander (non-Hispanic), other and unknown race, and missing race.

### 2.3. Procedure and Statistical Analyses

Chi square and T-tests were used to compare mean differences and proportions between the two groups: those with mTBI and those without mTBI for binary and continuous variables, respectively. Logistic regression models were used to compute the odds ratios (OR) for all three primary outcomes. For the length of stay variable, a count variable, we used generalized linear models with logarithmic link and Poisson distribution. Results from this model are presented in incidence rate ratios (IRRs) for ease of interpretation. All tests of significance were two-sided and the standard errors were bootstrapped to construct more precise confidence intervals for the coefficients.

## 3. Results

### 3.1. Descriptive Statistics

Summary statistics (see Table 1) showed that about 12% of the sample of ADSMs had a diagnosis of mTBI, based on a Glasgow coma scale score of 13–15. Sensitivity analyses using equivalent ICD-10 codes found similar results. For the total sample, about 3.5% of ADSMs were prescribed to be on “work duty limitations” following an outpatient visit or an inpatient admission. Further, among ADSMs who were prescribed to be on “work duty limitations”, a significant difference was found between those diagnosed with mTBI and those without any TBI (1% vs. 4%, *p* < 0.001). For the total sample, about 5% of ADSMs had any ED visits. A significant difference was found in ADSMs who had any ED visits between those diagnosed with mTBI and those without any TBI (1% vs. 6%, *p* < 0.001). For the total sample, about 4% of ADSMs had any inpatient admissions. Among ADSMs who had any inpatient admissions, a significant difference was found between those diagnosed with mTBI and those without any TBI (<1% vs. 4%, *p* < 0.001). The average length of stay was about 10 days for those with any inpatient admission. Additionally, for those with any inpatient admission, there was a significant difference in the average number of days stayed at the hospital between those diagnosed with mTBI compared to those without any TBI (22.5 days vs. 10.1 days, *p* = 0.0202).

Table 2 contains the summary statistics of the common symptoms of mTBI based on the ICD coding guidance from the Traumatic Brain Injury Center of Excellence. It also contains the prevalence for PTSD. For instance, PTSD is highly prevalent in this sample, with about 22% of individuals having a diagnosis of PTSD. However, for those with a diagnosis of mTBI, only about 8% of individuals in the sample had been diagnosed with PTSD compared to 24% for those without any TBI (*p* < 0.001).

### 3.2. Multivariate Results

#### 3.2.1. Commons Symptoms of mTBI and Work Functioning

Multivariate logistic regression results (see Table 3) showed odds of 0.11 (CI: 0.05–0.24, *p* < 0.01) for being “released with work duty limitations” and odds of 0.07 (CI: 0.02–0.24, *p* < 0.01) for having any ED visits for ADSMs with a diagnosis of mTBI compared to those with no diagnosis of TBI. Further, multivariate generalized linear models showed that the IRR of staying longer in an inpatient hospital is 4.48 (CI: 2.92–6.87, *p* < 0.01) times greater for those with mTBI compared to those with no diagnosis of TBI, holding all the covariates constant. Interacting mTBI diagnosis with cognitive and linguistic symptoms showed odds of 3.63 (CI: 1.40–9.36, *p* < 0.01) for being “released with work duty limitations” and odds of 4.98 (CI: 1.16–21.39, *p* < 0.05) for having any ED visits compared to those with no diagnosis of TBI and no cognitive and linguistic symptoms. Additionally, interacting mTBI diagnosis with sleep disturbance and chronic pain showed odds of 2.72 (CI: 1.31–5.65, *p* < 0.01) and odds of 11.56 (CI: 2.65–50.44, *p* < 0.01) for being “released with work duty limitations” compared to those with no diagnosis of TBI and none of these symptoms, respectively.

#### 3.2.2. Additional Results

For those with cognitive and linguistic symptoms, we found odds of 0.22 (CI: 0.13–0.37, *p* < 0.01) for having any ED visits and odds of 0.10 (CI: 0.03–0.40, *p* < 0.01) for having any inpatient admissions. Further, for those with any inpatient admissions, the IRR for staying longer in hospitals was 1.66 (CI: 1.11–2.48, *p* < 0.05) times greater for ADSMs with a history of deployment. ADSMs with a diagnosis of emotional and behavioral health issues had odds of 1.22 (CI: 1.06–1.41, *p* < 0.01) for having any ED visits, and higher odds of 3.51 (CI: 3.01–4.08, *p* < 0.01) for having any inpatient admissions compared to those with no emotional and behavioral health issues. Likewise, for those with inpatient admissions because of emotional and behavioral health issues, IRRs of 1.34 (CI: 1.18–1.52, *p* < 0.01) of staying longer in inpatient hospitals were found compared to those with no emotional and behavioral health issues, holding all the covariates constant.

Regression models also showed odds of 0.84 (CI: 0.69–1.02, *p* < 0.10) and odds of 0.69 (CI: 0.45–1.05, *p* < 0.10) for being “released with work duty limitations” for ADSMs with a reported diagnosis of sleep disturbances and those who experienced “other” categories of symptoms such as headache and chronic fatigue, compared to those without these symptoms, respectively. However, there were higher odds of 1.24 (CI: 1.11–1.40, *p* < 0.01) for having any ED visits and higher odds of 1.63 (CI: 1.36–1.95, *p* < 0.01) for having any inpatient admissions for ADSMs with a reported diagnosis of sleep disturbance compared to those without any sleep disturbance. Likewise, for those with inpatient admissions because of sleep disturbance, higher IRRs of 1.17 (CI: 1.02–1.33, *p* < 0.05) of staying longer in inpatient hospitals longer were found compared to those with no sleep disturbance, holding all the covariates constant. ADSMs with a reported diagnosis of emotional and behavioral health issues had higher odds of 1.22 (CI: 1.06–1.41, *p* < 0.01) for having any ED visits, and higher odds of 3.51 (CI: 3.01–4.08, *p* < 0.01) for having any inpatient admissions compared to those with no emotional and behavioral health issues. Likewise, for those with inpatient admissions because of emotional and behavioral health issues, IRRs of 1.34 (CI: 1.18–1.52, *p* < 0.01) of staying longer in inpatient hospitals were found compared to those with no emotional and behavioral health issues, holding all the covariates constant.

## 4. Discussion

Results from multivariate models showed that previously deployed ADSMs with a reported diagnosis of mTBI have lower odds of being “released with work duty limitations” and of having any ED visits compared to those with no reported diagnosis of TBI. That is, having a mTBI diagnosis is not a major factor that results in being “released with work duty limitations”. A plausible explanation is that as mTBI itself is not an “observable” condition, clinicians are more likely to decide to have an ADSM on work duty limitations on the basis of more observable symptoms such as PTSD, pain, sleep disturbance, irritability and anger, and attention and concentration deficits. We further explored the data and found that these symptoms are highly prevalent in this sample. Hence, the fact that our models controlled for these symptoms in a comprehensive way may allow us to tease out the distinct association between mTBI itself and work functioning, independent of these symptoms. For those with hospital inpatient admissions, an IRR of 4.48 times greater was found for staying longer in the hospital for patients with mTBI compared to those without TBI. In terms of the common symptoms of TBI, cognitive and linguistic, sleep disturbance and chronic pain showed higher odds for being “released with work duty limitations” for ADSMs, with a reported diagnosis of mTBI compared to those with no reported diagnosis of TBI and none of these symptoms. That is, mTBI experienced during deployment has significant effects on ADSMs’ work functioning through cognitive and linguistic, sleep disturbance and chronic pain symptoms. Because ADSMs are always screened for mTBI as well as these symptoms before and after any deployment, we are confident that the symptoms are a sequalae of mTBI and not pre-existing conditions. Further, these findings are consistent with other studies that have investigated the association between TBI and other employment-related outcomes [14,29,33,39,40,41,42,43,44,45,46,47].

Findings showed those with mTBI were less likely to have “work duty limitations” than those without mTBI. On the other hand, as the diagnosis of mTBI itself is complex, it is also possible that the negative association between the symptoms of sleep dysfunction, cognitive/linguistic deficits and pain with mTBI on work functioning may reflect different levels of injury for patients diagnosed with mTBI [48]. It is important to note that the reasons why some patients experience long-term sequelae in these domains, while others do not, is not well understood and requires further research. Further, there are no drugs currently available to treat TBI [49,50]. However, previous preclinical studies of drugs have shown positive signs when patients diagnosed with mTBI receive drug therapies for more than 12 h. Thus, future clinical trials may want to test the efficacy of drug therapies on functional outcomes such as return to work or recovery [50].

Findings from this study may have important implications. Many of the ADSMs in the study population are young and at a peak stage in their career development. Hence, these findings may inform and support a number of intervention strategies that target these symptoms to improve the quality of life and well-being of these ADSMs. Further, findings from this study may be relevant to rehabilitation planning and healthcare delivery across the continuum of recovery and occupational therapy practice. More specifically, rehabilitative interventions may focus on developing cognitive skills while reducing sleep disturbance and the level of pain to enhance work functioning or work productivity and readiness for these patients. These findings also inform the importance of supporting vocational programs and employment for patients diagnosed with mTBI in veterans’ affairs settings. Additionally, policymakers may want to increase the allocation of resources to preventive and rehabilitative programs and treatments that aim to address symptoms such as sleep dysfunction, cognitive/linguistic deficits and pain for patients diagnosed with mTBI in the MHS and veterans’ affairs, given the magnitude of these systems. Lastly, findings from this study will provide clinicians with additional information about the importance of developing clinical guidelines to prevent and treat these common symptoms of TBI in the MHS and they may also provide the foundation for assessing the cost-effectiveness of intervention and rehabilitation programs that target these specific symptoms of mTBI.

Despite the many strengths of this study, there are limitations that are noteworthy, specifically the lack of data on time since injury and duration of time off work. Further, our sample represents ADSMs with a history of deployment to Operation Enduring Freedom/Operation Iraqi Freedom, but we could not distinguish whether these ADSMs were exposed to combat nor to which operations they were deployed. These factors are important to understand injury level as well as symptoms severity. The validity of the measures used in this study may be of concern, particularly because of the reliance on ICD-10 codes from administrative claims data. However, as the MDR is a pristine dataset, there is confidence that the diagnoses used here are based on evidence-based assessments by healthcare providers in the MHS. Further, given the rigor of the study design, these factors provide credibility that work functioning or the workplace limitations are actually associated with the common symptoms of mTBI. Another strength of this study is the use of diagnosis codes that rely on accurate and comprehensive assessments by providers based on the clinical guidelines in the MHS, as these decisions have important implications for readiness in the MHS.

## 5. Conclusions

The study findings show that three common symptoms of mTBI—cognitive and linguistic, sleep disturbance and chronic pain—are the key mechanisms through which mTBI is associated with work functioning for ADSMs with a history of deployment. Further research is needed to understand why in particular it is these three common symptoms of mTBI. Future studies may also investigate the association between mTBI and duration of time off work to provide a comprehensive understanding of the effect of mTBI on work functioning in the MHS.

## Figures and Tables

**Table 1:** Summary statistics of dependent and socio-demographic variables, Military Health System data repository, 2015–2019.

Variables	Original Sample				Propensity Score Matched Sample		
	Total Sample (*n* = 22,995)	mTBI (*n* = 2673)	No TBI (*n* = 20,322)	*p*-Value	mTBI (*n* = 2671)	No TBI (*n* = 20,039)	*p*-Value
	Mean (95% CI)	Mean (95% CI)	Mean (95% CI)		Mean (95% CI)	Mean (95% CI)	
Dependent Variables							
Released w/limitations	0.035	0.0090	0.039	0.00	0.0090	0.038	
	[0.03, 0.04]	[0.01, 0.01]	[0.04, 0.04]		[0.01, 0.05]	[0.04, 0.04]	
Any Emergency Department Visit	0.050	0.0041	0.056	0.00	0.0041	0.054	
	[0.05, 0.05]	[0.00, 0.01]	[0.05, 0.06]		[0.00, 0.01]	[0.05, 0.06]	
Any In-patient Admissions	0.036	0.00075	0.041	0.00	0.00075	0.035	
	[0.03, 0.04]	[−0.00, 0.00]	[0.04, 0.04]		[−0.00, 0.00]	[0.04, 0.04]	
Key Independent Variables and Covariates							
mTBI ^#^	0.12				0.12	0.00	
	[0.11, 0.12]	[1.00, 1.00]	[0.00, 0.00]				
Age	35.5	36.8	35.3	0.00	36.8	36.8	0.994
	[35.38, 35.59]	[36.51, 37.08]	[35.21, 35.42]				
Female	0.12	0.048	0.13	0.00	REF	REF	REF
	[0.12, 0.12]	[0.04, 0.06]	[0.12, 0.13]				
Male	0.88	0.95	0.87	0.00	0.952	0.957	0.472
	[0.88, 0.88]	[0.94, 0.96]	[0.87, 0.88]				
White—Non-Hispanic	0.37	0.42	0.37	0.00	REF	REF	REF
	[0.37, 0.38]	[0.40, 0.44]	[0.36, 0.37]				
Black—Non-Hispanic	0.16	0.11	0.17	0.00	0.11	0.11	0.765
	[0.16, 0.17]	[0.10, 0.12]	[0.16, 0.17]				
Hispanic	0.056	0.063	0.055	0.09	0.06	0.05	0.059
	[0.05, 0.06]	[0.05, 0.07]	[0.05, 0.06]				
Asian Pacific Islander (Non-Hispanic)	0.026	0.020	0.027	0.02	0.02	0.02	0.846
	[0.02, 0.03]	[0.01, 0.03]	[0.02, 0.03]				
Other Unknown Race (Non-Hispanic)	0.13	0.11	0.14	0.00	0.11	0.12	0.393
	[0.13, 0.14]	[0.10, 0.13]	[0.13, 0.14]				
Race Missing	0.25	0.27	0.25	0.01	0.27	0.27	0.958
	[0.25, 0.26]	[0.26, 0.29]	[0.24, 0.26]				
Married	0.50	0.52	0.50	0.03	REF	REF	REF
	[0.50, 0.51]	[0.50, 0.54]	[0.49, 0.51]				
Single—Never Married	0.12	0.095	0.12	0.00	0.09	0.08	0.181
	[0.11, 0.12]	[0.08, 0.11]	[0.11, 0.12]				
Divorce, Separated, Widowed	0.050	0.043	0.051	0.05	0.04	0.03	0.053
	[0.05, 0.05]	[0.03, 0.05]	[0.05, 0.05]				
Unknown Marital Status	0.044	0.038	0.045	0.07	0.04	0.04	0.774
	[0.04, 0.05]	[0.03, 0.05]	[0.04, 0.05]				
Missing Marital Status	0.29	0.30	0.29	0.07	0.30	0.30	0.750
	[0.28, 0.29]	[0.29, 0.32]	[0.28, 0.29]				
Junior Enlisted	0.15	0.090	0.15	0.00	REF	REF	REF
	[0.14, 0.15]	[0.08, 0.10]	[0.15, 0.16]				
Senior Enlisted	0.69	0.73	0.68	0.00	0.73	0.75	0.022
	[0.68, 0.69]	[0.71, 0.74]	[0.67, 0.69]				
Junior Warrant Officer	0.10	0.11	0.10	0.41	0.11	0.10	0.258
	[0.10, 0.11]	[0.09, 0.12]	[0.10, 0.10]				
Senior Officer	0.066	0.078	0.065	0.01	0.08	0.07	0.082
	[0.06, 0.07]	[0.07, 0.09]	[0.06, 0.07]				
Army	0.80	0.75	0.80	0.00	REF	REF	REF
	[0.79, 0.80]	[0.74, 0.77]	[0.80, 0.81]				
Airforce	0.095	0.021	0.10	0.00	0.02	0.01	0.079
	[0.09, 0.10]	[0.02, 0.03]	[0.10, 0.11]				
Navy	0.041	0.13	0.030	0.00	0.13	0.10	0.001
	[0.04, 0.04]	[0.11, 0.14]	[0.03, 0.03]				
Marines	0.067	0.097	0.063	0.00	0.10	0.10	0.411
	[0.06, 0.07]	[0.09, 0.11]	[0.06, 0.07]				
Unknown Region	0.70	0.60	0.71	0.00	REF	REF	REF
	[0.70, 0.71]	[0.59, 0.62]	[0.71, 0.72]				
North Capital Region	0.034	0.13	0.021	0.00	0.13	0.13	0.571
	[0.03, 0.04]	[0.12, 0.14]	[0.02, 0.02]				
Tidewater	0.022	0.0015	0.025	0.00	0.00	0.00	0.706
	[0.02, 0.02]	[0.00, 0.00]	[0.02, 0.03]				
Fort Bragg	0.031	0.00037	0.035	0.00	0.00	0.00	1.000
	[0.03, 0.03]	[−0.00, 0.00]	[0.03, 0.04]				
Other Region	0.033	0.014	0.035	0.00	0.01	0.01	0.465
	[0.03, 0.04]	[0.01, 0.02]	[0.03, 0.04]				
Fort Jackson	0.026	0.036	0.025	0.00	0.04	0.03	0.103
	[0.02, 0.03]	[0.03, 0.04]	[0.02, 0.03]				
San Antonio	0.045	0.035	0.046	0.01	0.04	0.03	0.187
	[0.04, 0.05]	[0.03, 0.04]	[0.04, 0.05]				
Colorado Springs	0.044	0.095	0.038	0.00	0.10	0.07	0.000
	[0.04, 0.05]	[0.08, 0.11]	[0.04, 0.04]				
San Diego	0.027	0.075	0.020	0.00	0.07	0.07	0.914
	[0.02, 0.03]	[0.06, 0.08]	[0.02, 0.02]				
Puget Sound	0.037	0.0079	0.041	0.00	0.01	0.01	0.026
	[0.03, 0.04]	[0.00, 0.01]	[0.04, 0.04]				
Year 2015	0.028	0.021	0.029	0.02	REF	REF	REF
	[0.03, 0.03]	[0.02, 0.03]	[0.03, 0.03]				
Year 2016	0.27	0.27	0.27	0.78	0.27	0.31	0.004
	[0.26, 0.27]	[0.25, 0.29]	[0.26, 0.27]				
Year 2017	0.28	0.23	0.28	0.00	0.23	0.22	0.651
	[0.27, 0.28]	[0.21, 0.24]	[0.28, 0.29]				
Year 2018	0.24	0.24	0.24	0.74	0.24	0.23	0.658
	[0.24, 0.25]	[0.22, 0.26]	[0.24, 0.25]				
Year 2019	0.19	0.24	0.18	0.00	0.24	0.21	0.003
	[0.18, 0.19]	[0.23, 0.26]	[0.17, 0.19]				

mTBI = mild traumatic brain injuries; ^#^ mTBI: highest level of severity mild (Glasgow coma scale score 13–15).

**Table 2:** Summary statistics of common symptoms ^#^ of mild traumatic brain injury, Military Health System data repository, 2015–2019.

Variables	Original Sample				Propensity Score Matched Sample		
	Total Sample (*n* = 22,995)	mTBI (*n* = 2673)	No TBI (*n* = 20,322)	*p*-Value	mTBI (*n* = 2671)	No TBI (*n* = 20,039)	*p*-Value
	Mean (95% CI)	Mean (95% CI)	Mean (95% CI)		Mean (95% CI)	Mean (95% CI)	
Cognitive/Linguistic	0.059	0.25	0.034	0.00	0.24	0.27	0.022
	[0.06, 0.06]	[0.23, 0.26]	[0.03, 0.04]				
Hearing	0.010	0.043	0.0057	0.00	0.04	0.05	0.150
	[0.01, 0.01]	[0.03, 0.05]	[0.00, 0.01]				
Neurologic	0.041	0.20	0.020	0.00	0.20	0.20	0.612
	[0.04, 0.04]	[0.19, 0.22]	[0.02, 0.02]				
Emotional/Behavior	0.12	0.11	0.12	0.24	0.11	0.10	0.037
	[0.12, 0.13]	[0.10, 0.13]	[0.12, 0.13]				
Sleep Disturbance	0.23	0.24	0.23	0.16	0.24	0.22	0.092
	[0.22, 0.23]	[0.22, 0.25]	[0.22, 0.23]				
Vision	0.0062	0.027	0.0035	0.00	0.03	0.03	0.055
	[0.01, 0.01]	[0.02, 0.03]	[0.00, 0.00]				
Other	0.0017	0.00075	0.0018	0.09	0.00	0.00	0.414
	[0.00, 0.00]	[−0.00, 0.00]	[0.00, 0.00]				
Post-Traumatic Stress Disorder	0.22	0.083	0.24	0.00	0.08	0.08	0.516
	[0.22, 0.23]	[0.07, 0.09]	[0.23, 0.24]				
Pain	0.012	0.0071	0.012	0.01	0.01	0.01	0.612
	[0.01, 0.01]	[0.00, 0.01]	[0.01, 0.01]		[0.00, 0.01]	[0.01, 0.01]	
mTBI * Cognitive/Linguistic	0.029	0.25	0		0.25	0	
	[0.03, 0.03]	[0.23, 0.26]	[0.00, 0.00]		[0.23, 0.26]	[0.00, 0.00]	
mTBI * Hearing	0.0050	0.043	0		0.043	0	
	[0.00, 0.01]	[0.03, 0.05]	[0.00, 0.00]		[0.03, 0.05]	[0.00, 0.00]	
mTBI * Neurological	0.024	0.20	0		0.20	0	
	[0.02, 0.03]	[0.19, 0.22]	[0.00, 0.00]		[0.19, 0.22]	[0.00, 0.00]	
mTBI * Emotional/Behavioral	0.013	0.11	0		0.11	0	
	[0.01, 0.01]	[0.10, 0.13]	[0.00, 0.00]		[0.10, 0.13]	[0.00, 0.00]	
mTbi * Sleep	0.028	0.24	0		0.24	0	
	[0.03, 0.03]	[0.22, 0.25]	[0.00, 0.00]		[0.22, 0.25]	[0.00, 0.00]	
mTBI * Vision	0.0031	0.027	0		0.027	0	
	[0.00, 0.00]	[0.02, 0.03]	[0.00, 0.00]		[0.02, 0.03]	[0.00, 0.00]	
mTBI * Other	0.000087	0.00075	0		0.00075	0	
	[−0.00, 0.00]	[−0.00, 0.00]	[0.00, 0.00]		[−0.00, 0.00]	[0.00, 0.00]	
mTBI * Post-Traumatic Stress Disorder	0.0096	0.083	0		0.083	0	
	[0.01, 0.01]	[0.07, 0.09]	[0.00, 0.00]		[0.07, 0.09]	[0.00, 0.00]	
mTBI * Pain	0.00083	0.0071	0		0.0071	0	
	[0.00, 0.00]	[0.00, 0.01]	[0.00, 0.00]		[0.00, 0.01]	[0.00, 0.00]	

mTBI = mild traumatic brain injuries; ^#^ These common symptoms of TBI are based on the ICD coding guidance from the Traumatic Brain Injury Center of Excellence (accessed 23 October 2020 at https://dvbic.dcoe.mil/cogrehab/pdf/dvbic_4383_icd-10-coding-guidance-tbi_v1.4_2017–09-06_508.pdf).

**Table 3:** Regression results for multivariate models for servicemembers with a history of deployment, Military Health System data repository, 2015–2019.

Variables	Logistic Regression Models			Generalized Linear Models
	(*n* = 22,710)	(*n* = 22,710)	(*n* = 22,710)	(*n* = 696)
	O.R. (95% CI)	O.R. (95% CI)	O.R. (95% CI)	I.R.R. (95% CI)
	Released w/Limitations	Emergency Department Visit	Inpatient Admission	Length of Stay
mTBI ^#^	0.11 ***	0.07 ***	0.00	4.48 ***
	[0.05, 0.24]	[0.02, 0.24]	[0.00, 4.27 × 10^12^]	[2.92, 6.87]
Cognitive/Linguistic	0.72	0.22 ***	0.10 ***	1.66 **
	[0.42, 1.21]	[0.13, 0.37]	[0.03, 0.40]	[1.11, 2.48]
Hearing	0.81	1.37	2.42	1.06
	[0.32, 2.08]	[0.76, 2.50]	[0.83, 7.07]	[0.73, 1.55]
Neurologic	0.99	1.48	1.63	1.26
	[0.56, 1.74]	[0.91, 2.42]	[0.88, 3.03]	[0.94, 1.70]
Emotional/Behavioral	0.95	1.22 ***	3.51 ***	1.34 ***
	[0.76, 1.19]	[1.06, 1.41]	[3.01, 4.08]	[1.18, 1.52]
Sleep	0.84 *	1.24 ***	1.63 ***	1.17 **
	[0.69, 1.02]	[1.11, 1.40]	[1.36, 1.95]	[1.02, 1.33]
Vision	0.68	0.63	1.09	1.01
	[0.19, 2.47]	[0.22, 1.83]	[0.32, 3.73]	[0.45, 2.27]
Other	0.69 *	1.62 ***	2.27 ***	1.45 ***
	[0.45, 1.05]	[1.17, 2.25]	[1.44, 3.59]	[1.16, 1.82]
Post-Traumatic Stress Disorder	1.00	1.00	1.00	1.00
	[1.00, 1.00]	[1.00, 1.00]	[1.00, 1.00]	[1.00, 1.00]
Pain	1.18	0.13 ***	1.88 *	1.16
	[0.52, 2.70]	[0.04, 0.43]	[0.99, 3.59]	[0.86, 1.57]
mTBI * Cognitive/Linguistic	3.63 ***	4.98 **	---	---
	[1.40, 9.36]	[1.16, 21.39]	---	---
mTBI * Hearing	0.75	---	6.22	0.68
	[0.19, 2.94]	---	[0.00, 8.99 × 10^10^]	[0.33, 1.37]
mTBI * Neurological	1.51	0.23 ***	---	---
	[0.52, 4.44]	[0.10, 0.55]	---	---
mTBI * Emotional/Behavioral	1.11	1.72	---	---
	[0.55, 2.24]	[0.37, 8.02]	---	---
mTBI * Sleep	2.72 ***	0.98	---	---
	[1.31, 5.65]	[0.25, 3.90]	---	---
mTBI * Post-Traumatic Stress Disorder	1.00	---	1.00	---
	[1.00, 1.00]	---	[1.00, 1.00]	---
mTBI * Pain	11.56 ***	---	136.08	---
	[2.65, 50.44]	---	[0.00, 1.25 × 10^12^]	---
Age	1.02	0.92 *	1.01	1.19 ***
	[0.92, 1.12]	[0.83, 1.01]	[0.91, 1.12]	[1.10, 1.29]
Age Squared	1.00	1.00	1.00	1.00 ***
	[1.00, 1.00]	[1.00, 1.00]	[1.00, 1.00]	[1.00, 1.00]
Male	0.98	1.35 ***	1.12	1.28 **
	[0.83, 1.16]	[1.12, 1.62]	[0.90, 1.41]	[1.04, 1.56]
Black—Non-Hispanic	0.97	1.18 **	0.90	0.98
	[0.78, 1.21]	[1.03, 1.35]	[0.72, 1.13]	[0.81, 1.18]
Asian Pacific Islander (Non-Hispanic)	0.89	1.32 *	0.75	0.57 **
	[0.57, 1.40]	[0.98, 1.78]	[0.42, 1.33]	[0.34, 0.96]
Hispanic	0.88	1.49 ***	1.51 ***	0.79 *
	[0.60, 1.30]	[1.18, 1.89]	[1.13, 2.02]	[0.62, 1.00]
Other Unknown Race (Non-Hispanic)	1.07	0.84	1.23 *	0.78 **
	[0.88, 1.31]	[0.64, 1.11]	[0.97, 1.57]	[0.65, 0.95]
Race Missing	1.73 ***	0.88	0.11 ***	1.35 **
	[1.18, 2.53]	[0.68, 1.15]	[0.06, 0.18]	[1.04, 1.76]
Single—Never Married	1.08	0.84 *	1.64 ***	1.04
	[0.85, 1.36]	[0.69, 1.02]	[1.29, 2.09]	[0.88, 1.23]
Divorce, Separated, Widowed	0.83	1.07	1.21	1.40 ***
	[0.53, 1.30]	[0.77, 1.49]	[0.88, 1.65]	[1.13, 1.74]
Unknown Marital Status	1.01	0.66 *	0.48 ***	0.90
	[0.76, 1.35]	[0.41, 1.05]	[0.29, 0.81]	[0.57, 1.42]
Missing Marital Status	0.71 **	1.16	---	---
	[0.51, 0.97]	[0.87, 1.55]	---	---
Senior Enlisted	0.81	1.09	0.85	0.90
	[0.62, 1.04]	[0.84, 1.40]	[0.70, 1.04]	[0.72, 1.12]
Junior Warrant Officer	0.55 ***	1.46 ***	0.73 *	1.25
	[0.37, 0.82]	[1.14, 1.87]	[0.50, 1.06]	[0.94, 1.66]
Senior Officer	0.50 ***	1.33	0.67	0.88
	[0.32, 0.79]	[0.88, 2.01]	[0.40, 1.14]	[0.59, 1.30]
Airforce	1.28	0.49 ***	0.22 ***	0.70 *
	[0.93, 1.77]	[0.42, 0.57]	[0.13, 0.36]	[0.46, 1.07]
Navy	1.49 **	0.51 ***	1.02	0.75
	[1.02, 2.18]	[0.35, 0.73]	[0.69, 1.52]	[0.53, 1.07]
Marines	1.75 ***	0.32 ***	0.62 ***	0.49 ***
	[1.28, 2.40]	[0.21, 0.47]	[0.43, 0.89]	[0.34, 0.71]
North Capital Region	0.63	0.88	17.91 ***	1.39 ***
	[0.31, 1.29]	[0.51, 1.52]	[13.27, 24.18]	[1.14, 1.71]
Tidewater	1.05	0.03 ***	1.66	0.86
	[0.61, 1.83]	[0.01, 0.08]	[0.73, 3.76]	[0.55, 1.34]
Fort Bragg	1.42 *	0.94	---	---
	[0.95, 2.12]	[0.70, 1.26]	---	---
Other Region	0.50 ***	0.29 ***	0.36 ***	1.04
	[0.33, 0.74]	[0.21, 0.42]	[0.17, 0.75]	[0.58, 1.86]
Fort Jackson	1.22	0.34 ***	0.75	1.50 ***
	[0.71, 2.09]	[0.19, 0.59]	[0.45, 1.25]	[1.15, 1.96]
San Antonio	0.91	0.06 ***	0.34 **	0.55
	[0.57, 1.45]	[0.02, 0.16]	[0.14, 0.80]	[0.26, 1.13]
Colorado Springs	0.45 ***	0.44 ***	5.71 ***	0.48 ***
	[0.30, 0.66]	[0.28, 0.70]	[4.45, 7.33]	[0.39, 0.58]
San Diego	1.16	0.92	2.24 **	0.66 *
	[0.80, 1.68]	[0.47, 1.83]	[1.10, 4.56]	[0.41, 1.05]
Puget Sound	0.62	0.52 ***	---	---
	[0.34, 1.14]	[0.37, 0.74]	---	---
Year 2016	1.57 *	0.69 **	1.35	1.23
	[0.94, 2.63]	[0.48, 0.99]	[0.81, 2.25]	[0.73, 2.07]
Year 2017	1.66 *	0.69 **	1.56	1.68 *
	[1.00, 2.75]	[0.49, 0.99]	[0.91, 2.65]	[1.00, 2.81]
Year 2018	1.68 **	0.50 ***	1.79 **	2.10 ***
	[1.04, 2.73]	[0.35, 0.72]	[1.10, 2.89]	[1.26, 3.51]
Year 2019	1.75 *	0.41 ***	1.65 *	2.05 ***
	[0.99, 3.10]	[0.28, 0.60]	[0.96, 2.83]	[1.22, 3.44]

* *p* < 0.10; ** *p* < 0.05; *** *p* < 0.01. mTBI = mild traumatic brain injuries; I.R.R. = incidence rate ratios; ^#^ mTBI: highest level of severity mild (Glasgow coma scale score 13–15).

## Data Availability

All retrospective outpatient and inpatient data from 2015 to 2019 are available in the Military Health System Data Repository (MDR).
